# Abundant chaos in a mixer model with a hysteretic iron core inductance

**DOI:** 10.1038/s41598-023-48046-z

**Published:** 2023-12-07

**Authors:** M. Aminou, U. Simo Domguia, S. A. Oumarou, P. Woafo

**Affiliations:** 1 Carnot Energy Laboratory (LEC), Faculty of Sciences, University of Bangui, Box 908, Bangui, Central African Republic; 2 Department of Mechanical Engineering, Faculty of Engineering and Technology (FET), University of Buea, P.O. Box 63, Buea, Cameroon; 3 Laboratory on Modelling and Simulation in Engineering, Biomimetics and Prototypes, and TWAS Research Unit, Faculty of Science, University of Yaounde I, Box 812, Yaounde, Cameroon; 4 Laboratory of Products Development and Entrepreneurship, Institut Superieur de l’Innovation et de Technologie, P.O. Box 8210, Yaounde, Cameroon

**Keywords:** Physics, Statistical physics, thermodynamics and nonlinear dynamics

## Abstract

Industrial mixers are equipment used in food, drug, chemical and semiconductor industries. Chaotic mixing has been proposed to improve the degree of homogeneity and reduce the energy consumption. This paper deals with dynamical studies of a mixer model with complex rotational movements. The complexity is generated by an inductance with hysteretic characteristics. Mathematical methods and numerical simulations are used to display the different dynamical states which are period-nT, pulse, bursting and chaotic signals. Good agreement is found between the mathematical and numerical results. In general, it is found that chaos is highly abundant in the model.

## Introduction

Electromechanical devices are frequently used in a number of industrial and household applications such as shaking, sieving, mixing,… to name just some few examples^1–6^. These systems offer several advantages due to the mechanical power they provide for the accomplishment of several specific tasks. The industrial importance of mixing can hardly be exaggerated. Chemical, petrochemical, and pharmaceutical processes usually require bringing reactants into close contact by imposing a mixing flow^7–9^.

In recent years, chaotic mixing has been proposed to improve the energy efficiency, the degree of homogeneity and the duration of the mixing process^10–14^. The chaos is generated using either geometrically asymmetric design of the mixer to produce a practical chaotic motion and feedback action.

Instead of using mechanical means or feedback, our idea is to produce the desired chaotic motion electrically using a RLC series circuit with hysteretic iron-core inductor. While exceptionally simple passive elements such as resistors, capacitors, and air core inductors do respond to a first order approximation nearly linearly, in devices that have ferromagnetic cores, the relationship between the flux density and magnetic field strength in the core is nonlinear^15,16^. This nonlinear relationship depends on several factors among which the chemical constitution and structure of the magnetic material, the technological process for its fabrication, and the way the material is used. The nonlinear characteristics of magnetic materials exhibits hysteresis^17,18^.

In most cases, instruments such as blender, mixer, drill, vacuum cleaner, washing machine, etc., contain a DC motor. For this type of motor, the excitation winding is connected in series with the rotor winding^19–21^. This type of motor can work with either direct current (DC) or alternating current (AC)^22–24^. The speed of the universal motor can be very high, and necessarily depends on the load torque, also called disturbance torque, and the supply voltage. Indeed, the torque of a device such as an electric mixer depends on the different applications.

Many researchers contributed to the better understanding of chaotic motors for industrial mixers^25–30^. The obtained results indicate that chaotic mixing prevents the formation of segregated regions, thus leading to efficient mixing compared with normal constant speed mixing. Starting from the 1990’s, a number of research activities on chaos in motors have been carried out. Most of them are based on the identification of chaos^28^, the avoidance of chaos^29^ and the stabilization of chaos^30^ in various types of electric motors. Rather than negatively avoiding the occurrence of chaos in motors, the chaotization of the DC motor (the agitator) using time-delay feedback control was firstly proposed and implemented for use in industrial mixers^31^. Compared with the mechanical means, the electrically implemented chaotic motion motor not only produces the desired chaotic mixing, but also offers the advantages of high flexibility and high controllability.

More studies on chaotic motors are still required due to their potential applications. This justifies the study conducted in this work. Its goal is to model and study the behavior of a motor actuated by a RLC series circuit with hysteretic iron-core inductor where one needs to transfer the chaotic behavior of nonlinear electric circuit on the motor. Indeed, the results presented in refs^31,32^ show the possibility of obtaining chaotic behaviors on a motor when powered by a DC voltage and their application for chaotic mixing^33^. It is of special importance to mention that it has been experimentally demonstrated that the chaotic mixing has two main advantages: high reduction of the duration of the mixing process, but also reduction of the energy consumed for the mixing process as compared to motors functioning with a constant speed^25,27,33^. Guided by these initial studies, we focus on the response of a mixer when powered by an alternating source. A description of the model is given. The modelling equations of the device are then derived showing a set of two ordinary differential equations (the electric circuit) coupled to an ordinary differential equation given by the Newton law (that of the motor).

The outline of the paper is as follows. “Mixer model: description, modelling and amplitudes of period-1T oscillations” section deals with the presentation and modelling of the device powered by an alternating source generator. “Bifurcation diagrams and frequency response curves” section presents the mathematical and numerical results obtained when the motor is powered by a sine voltage. Numerical results in the case where the device is powered by a square signal are given in “Mixer model powered by a square signal” section. The work is concluded with some remarks and future prospects in “Conclusion” section.

## Mixer model: description, modelling and amplitudes of period-1T oscillations

### Description of the device

**Figure 1:**
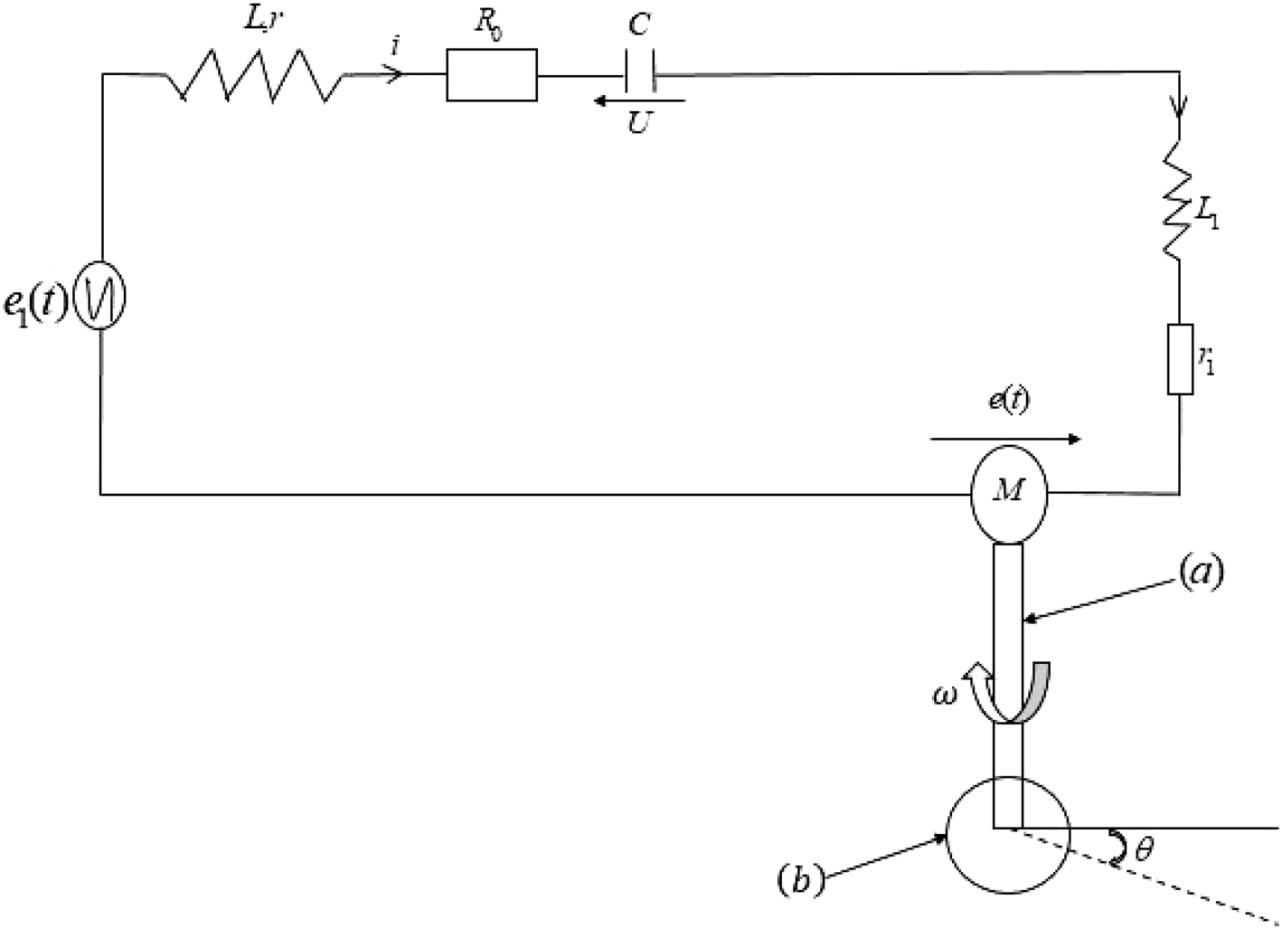
Mixer driving an utensil.

The system studied is presented in Fig. 1. It comprises a motor made of two main parts: the stator and the rotor connected in series. The motor is powered by an alternating voltage source.

The stator is a $RLC$ circuit comprising an inductor with a ferromagnetic core. $L$ is the inductance of this coil, $r$ is the internal resistance of the inductor and $R_{0}$ is an additional resistance used to monitor the magnitude of the current through the inductor and to measure the current through the circuit. $C$ is the capacitance of the capacitor of the circuit.

The electric part of the motor is a $L_{1} r_{1}$ circuit where $L_{1}$ is the inductance of the coil constituting the rotor and $r_{1}$ its internal resistance. The mixer consists of a single block from the motor up to the mixer foot (a), at which one can attach various accessories (utensils) (b) thanks to an open dome. We consider in our study that the foot of mixer is nothing other than the motor shaft, which transmits the movement to the disc representing any arm.

### Mathematical modelling

#### Equation of the electrical part

Applying Kirchhoff’s laws to Fig. 1, and because the inductance of the inductor used in RLC circuit is not constant, the state equations describing the electrical part are given below:

1 $$ \left\{ \begin{aligned} & \frac{d(Li)}{{dt}} + L_{1} \frac{di}{{dt}} + Ri - U + e(t) = E_{m} \sin (\omega_{e} t) \\ & i = - C\frac{dU}{{dt}} \\ \end{aligned} \right. $$

where $R$ is the total resistance of the circuit given by $R = R_{0} + r + r_{1}$, $U$ the voltage across the capacitor, $i$ the current through the circuit, $E_{m}$ the magnitude of the external voltage and $e(t)$ is the induced back electromotive force. The inductance of the inductor containing the ferromagnetic material is given by the mathematical expression (2)^17^:

2 $$ L = \frac{{\mu_{0} N^{2} A}}{l}\; + \frac{{B_{S} NA}}{i}\tanh \left( {\frac{\alpha Ni}{{2l}} - \frac{\sigma }{2}\;} \right)\;{\text{with}}\;\sigma = \beta sign\left( {\frac{di}{{dt}}} \right) $$

where $B_{s}$ is the saturation flux density. *A* and $l$ are respectively the cross-sectional areas and the average lengths of the ferromagnetic material. $N$ is the number of turns (windings), $\mu_{0}$ is the permeability of the vacuum. The parameters $\alpha$ and $\beta$ are constants depending on the remanent and coercice magnetic fields.

The induced back-electromotive force is proportional to the motor speed with expression given as:

3 $$ e(t) = K_{e} \omega (t) $$

where $K_{e}$ is the back-electromotive force (back-EMF) constant and $\omega$ the rotational speed of the rotor. By replacing Eqs. (2) and (3) in (1), the equations of the electrical part are given as follows:

4 $$ \left\{ \begin{aligned} & \left\{ {1 - \eta \left[ {\frac{{ - 1 + \cosh \left( {\frac{\alpha Ni}{l} - \sigma } \right)}}{{1 + \cosh \left( {\frac{\alpha Ni}{l} - \sigma } \right)}}} \right]} \right\}\frac{di}{{dt}} + \frac{R}{{L_{0} }}i - \frac{U}{{L_{0} }} + \frac{{K_{e} }}{{L_{0} }}\omega (t) = \frac{{E_{m} }}{{L_{0} }}\sin (\omega_{e} t) \\ & i = - C\frac{dU}{{dt}} \\ \end{aligned} \right. $$

5 $$ {\text{with}}\;L_{0} = \frac{{\mu_{0} N^{2} A}}{l}\left( {1 + \frac{{\alpha B_{s} }}{{2\mu_{0} }}} \right) + L_{1} \;{\text{and}}\;\eta = \frac{{\alpha B_{s} N^{2} A}}{{2lL_{0} }} $$

#### Equation of the mechanical part

By application of the fundamental relation of the dynamics of motion, the mechanical part is described by the following equation:

6 $$ J\frac{d\omega (t)}{{dt}} = - f_{1} \omega (t) - C_{r} (t) + K_{m} i(t) $$

where $J$ is the load moment of inertia (of the rotor axis or of the mixer taking into account the presence of the motor shaft $J_{m}$ and the inertia of the utensils $J_{u}$). $C_{r}$ is the load torque, which is an additional torque due to the presence of the material to be mixed or cut by the mixer (it will be neglected in this study assuming that the viscous damping term accounts also for the resistance due to the product). $f_{1}$ is the viscous damping coefficient and $K_{m}$ is the electromechanical torque constant. Thus, the equation of the mechanical part is governed by:

7 $$ J\frac{d\omega (t)}{{dt}} = - f_{1} \omega (t) + K_{m} i(t) $$

#### Dimensionless equations

The following dimensionless variables are used:

8 $$ t = \frac{\tau }{{\omega_{0} }},\;\;i = I_{0} x,\;\;U = y,\;\;\omega = \omega_{1} z $$

where $\omega_{0}$ is a reference frequency, $\omega_{1}$ is the reference parameter of the angular rotation speed of the motor and $I_{0}$ is the reference parameter of the current given by $I_{0} = \frac{l}{\alpha N}$. By replacing the variables of Eq. (8) in Eqs. (4) and (7), we thus obtain the dimensionless equations of the mixer given below:

9 $$ \left\{ \begin{aligned} & \left[ {(1 + \eta ) + (1 - \eta )\cosh \left( {x - \sigma } \right)} \right]\dot{x} = \left[ { - \lambda x + \varepsilon y - \gamma z + E\sin (\Omega \tau )} \right]\left[ {1 + \cosh \left( {x - \sigma } \right)} \right] \\ & \dot{y} = - qx \\ & \dot{z} = - \delta_{1} z + \delta_{3} x \\ \end{aligned} \right. $$

with the following dimensionless coefficients:

10 $$  \begin{aligned}    & \lambda  = \frac{R}{{L_{0} \omega _{0} }},\;\varepsilon  = \frac{1}{{L_{0} \omega _{0} I_{0} }},\;E = \frac{{E_{m} }}{{L_{0} \omega _{0} I_{0} }},\;q = \frac{{I_{0} }}{{C\omega _{0} }},\;\Omega  = \frac{{\omega _{e} }}{{\omega _{0} }} \\     & \gamma  = \frac{{K_{e} \omega _{1} }}{{L_{0} \omega _{0} I_{0} }},\;\delta _{1}  = \frac{{f_{1} }}{{J\omega _{0} }},\;\delta _{3}  = \frac{{K_{m} I_{0} }}{{J\omega _{1} \omega _{0} }} \\  \end{aligned}   $$

### Mathematical analysis and expression of the oscillations amplitude

As simplifying condition, let parameter $\sigma$ be neglected. Equation (9) takes the reduced form:

11 $$ \left\{ \begin{aligned} & \left[ {(1 + \eta ) + (1 - \eta )\cosh \left( x \right)} \right]\dot{x} = \left[ { - \lambda x + \varepsilon y - \gamma z + E\sin (\Omega \tau )} \right]\left[ {1 + \cosh \left( x \right)} \right] \\ & \dot{y} = - qx \\ & \dot{z} = - \delta_{1} z + \delta_{3} x \\ \end{aligned} \right. $$

Using the formalism developed in Nana et al.^17^, the approximate oscillatory states of period-T are expressed as

12 $$ x = X_{1m} \cos (\Omega \tau + \varphi_{1} ),\;\;y = X_{2m} \cos (\Omega \tau + \varphi_{2} )\;\;{\text{and}}\;\;z = X_{3m} \cos (\Omega \tau + \varphi_{3} ) $$

where X_im_ and ϕ_i_ are respectively the amplitudes and initial phases. The amplitudes satisfy the following equations:

13 $$ a_{3} X_{1m}^{3} + a_{2} X_{1m}^{2} + a_{1} X_{1m}^{{}} + a_{0} = 0,\;\;X_{2} = \frac{q}{\Omega }X_{1} ,\;\;{\text{and}}\;\;X_{3} = \frac{{\delta_{3} }}{{\sqrt {\Omega^{2} + \delta_{1}^{2} } }}X_{1} $$

The coefficients of the above equation are defined as:

14 $$ \left\{ \begin{aligned} & a_{3} = 4\Omega^{4} \left[ {2\gamma \varsigma_{3} + 2\eta - 4 - \left( {\gamma \varsigma_{3} + \eta } \right)^{2} } \right] - \Omega^{2} \left[ {\left( {\gamma \varsigma_{2} + \lambda } \right)^{2} + 8\varepsilon q(\gamma \varsigma_{3} + \eta + 1)} \right] - 4\varepsilon^{2} q^{2} \\ & a_{2} = - 64\Omega^{4} (\gamma \varsigma_{3} - 1)(\gamma \varsigma_{3} + \eta - 1) - \Omega^{2} \left[ {128\varepsilon q(\gamma \varsigma_{3} + \frac{\eta }{4} - 1) + 32\gamma \varsigma_{2} (\gamma \varsigma_{2} + 2\lambda ) + 32\lambda^{2} - E^{2} } \right] \\ & - 64\varepsilon^{2} q^{2} \\ & a_{1} = - 256\Omega^{4} (\gamma \varsigma_{3} - 1)^{2} + 32\Omega^{2} \left[ {16\varepsilon q( - \gamma \varsigma_{3} + 1) - 8\gamma \varsigma_{2} (\gamma \varsigma_{2} + 2\lambda ) - 8\lambda^{2} + E^{2} } \right] - 256\varepsilon^{2} q^{2} \\ & a_{0} = 256\Omega^{2} E^{2} \\ \end{aligned} \right. $$

The equation in X_im_ can be solved mathematically or numerically and thus one will have the frequency response curves for all X_im_ (this will be presented below along with the results of the direct numerical simulation).

## Bifurcation diagrams and frequency response curves

In this section, we present both the analytical and numerical simulation. The analytical results have been obtained using Eq. (13) and the numerical simulation is applied on Eq. (11). The numerical simulation uses the fourth-order Runge–Kutta algorithm with a time step $\Delta t = 0.01$ and with (0,0,0) as initial conditions.

The parameters of the electrical part are as follows:

$$ \begin{aligned} & {\text{r}} = {8}\;\Omega ,\;{\text{r}}_{{1}} = {7}\;\Omega ,\;{\text{l}} = {24}\;{\text{cm}},\;{\text{A}} = {176}.{71}\;{\text{mm}}^{{2}} ,\;{\text{B}}_{{\text{s}}} = {13}0\;{\text{mT}},\;{\text{N}} = {2}000, \\ & \quad {\text{L}}_{{1}} = {1}.{5}\;{\text{mH}},\;{\text{C}} = {4}.{11}\;\mu {\text{F}}, \;{\text{I}}_{0} = {13}.{6}\;{\text{mA}},\;\alpha = {88}.{23} \times {1}0^{{ - {4}}} \;{\text{m}}/{\text{A}}, \\ & \quad \beta = {88}.{42} \times {1}0^{{ - {2}}} ,\;{\text{L}}_{0} = {1694}.{22}\;{\text{mH}},\;\eta = 0.{9969}. \\ \end{aligned} $$

R_0_ is variable.

Those of the mechanical part used are given below:

$$ {\text{K}}_{{\text{c}}} = 0.00{1}\;{\text{N}}\;{\text{m}}\;{\text{rpm}},\;{\text{K}}_{{\text{e}}} = 0.0{4975}\;{\text{S}}\;{\text{V}}/{\text{rad}}/{\text{A}},\;{\text{K}}_{{\text{m}}} = 0.0{4998}\;{\text{S}}\;{\text{V}}/{\text{rad}}/{\text{A}},\;{\text{J}} = {1}.0{38} \times {1}0^{{ - {6}}} {\text{kg}}\;{\text{m}}^{{2}} . $$

The damping coefficient f_1_ is variable.

The resistance R_0_, the damping coefficient f_1_, the amplitude $E_{m}$ of the voltage and the voltage frequency $\omega_{e} = 2\pi f$ are used as control parameters. The dimensionless coefficients are obtained as follows:

$$ \varepsilon = {43}.{39} \times {1}0^{{ - {2}}} ,\;\gamma = {2}.{15} \times {1}0^{{ - {2}}} ,\;{\text{q}} = {33}.0{9},\;\delta_{{3}} = {6}.{544} $$

The coefficients λ, δ_1_, E and Ω are the control parameters.

### Effect of the resistance $R_{0}$

**Figure 2:**
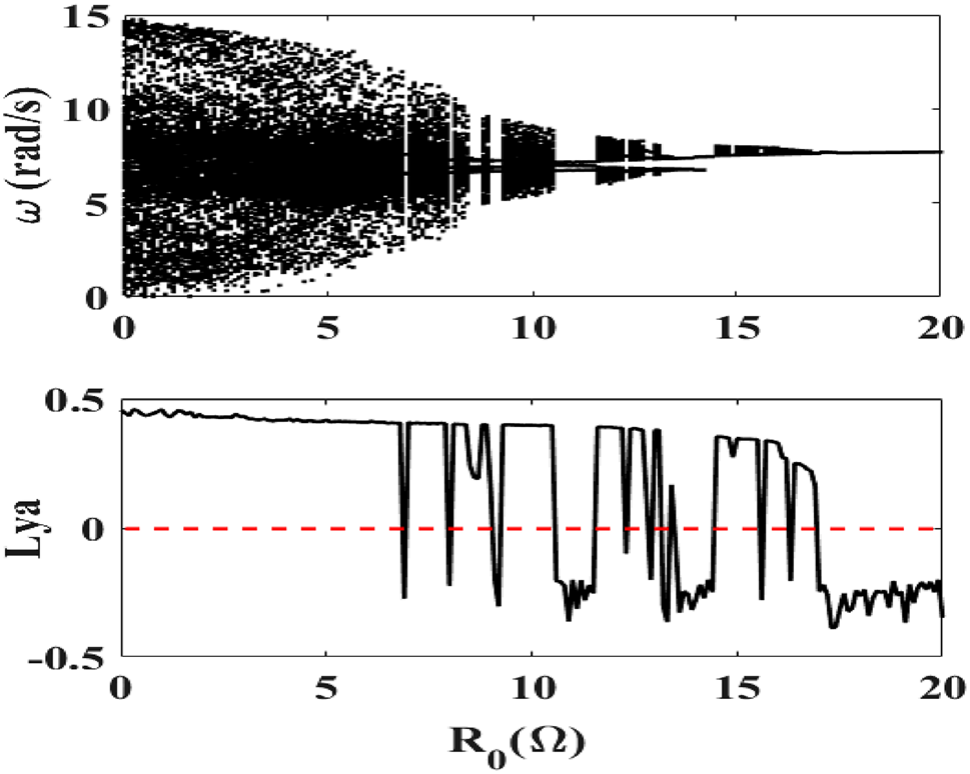
Bifurcation diagram of the motor speed and its Lyapunov exponent for $f = 50\;{\text{Hz}}$ and $0 \, \Omega \le R_{0} \le 20 \, \Omega$ with λ = 11.81 × 10^−2^ and δ_1_ = 1.925, $E_{m} = 40\;{\text{V}}$.

We analyze here the behavior of the motor speed under the variation of the value of the resistance. Figure 2 shows the bifurcation diagram of the mechanical part and its Lyapunov exponent when the resistance $R_{0}$ varies. The bifurcation diagram shows chaotic behavior of the device which is confirmed by its Lyapunov exponent when the resistance varies in several domains. One observes that the transitions from periodic behaviors (corresponding to negative values of the Lyapunov exponent) to chaos (corresponding to positive Lyapunov exponent) are quite abrupt.

### Effect of the viscous damping coefficient

**Figure 3:**
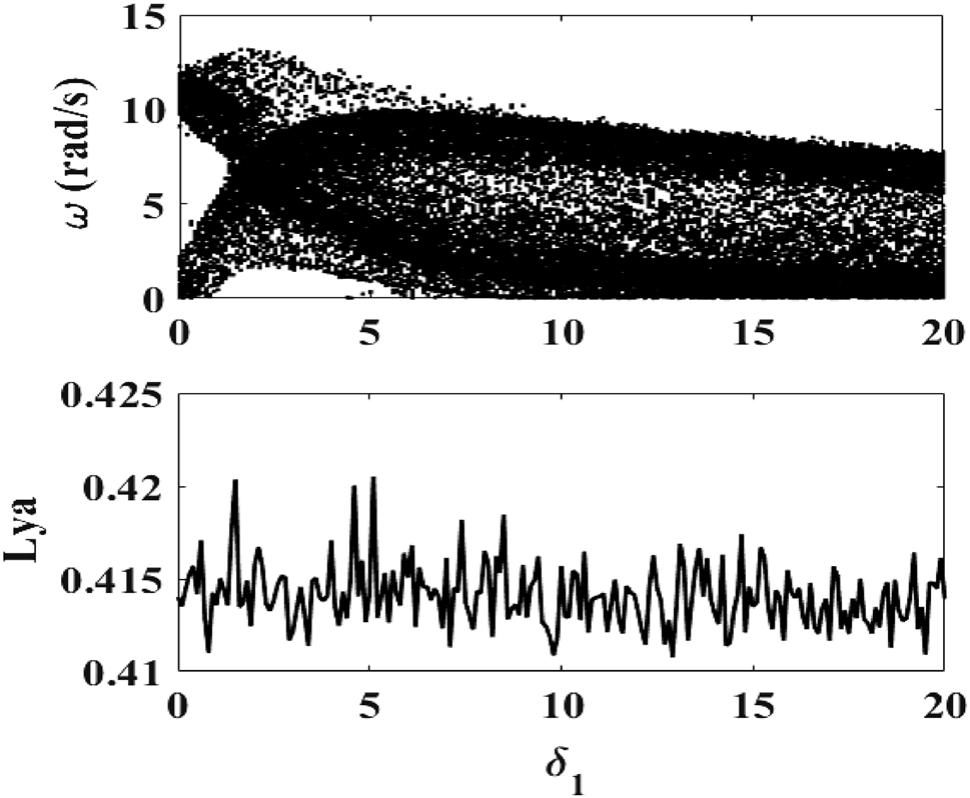
Bifurcation diagram of the motor speed and its Lyapunov exponent for $f = 50\;{\text{Hz}}$, $R_{0} = 5 \, \Omega$ and $0 \le \delta_{1} \le 20$, $E_{m} = 40\;{\text{V}}$, $R_{0} = 5 \, \Omega$ (λ = 11.81 × 10^−2^).

Figure 3 below presents the bifurcation diagram of the mechanical part and its Lyapunov exponent when the parameter $\delta_{1}$ (linked to the viscous damping coefficient) varies. The bifurcation diagram of Fig. 3 shows chaos in the whole range of the variation of $\delta_{1}$. This is also confirmed by the variation of the Lyapunov exponent.

### Effects of the amplitude $E_{m}$

**Figure 4:**
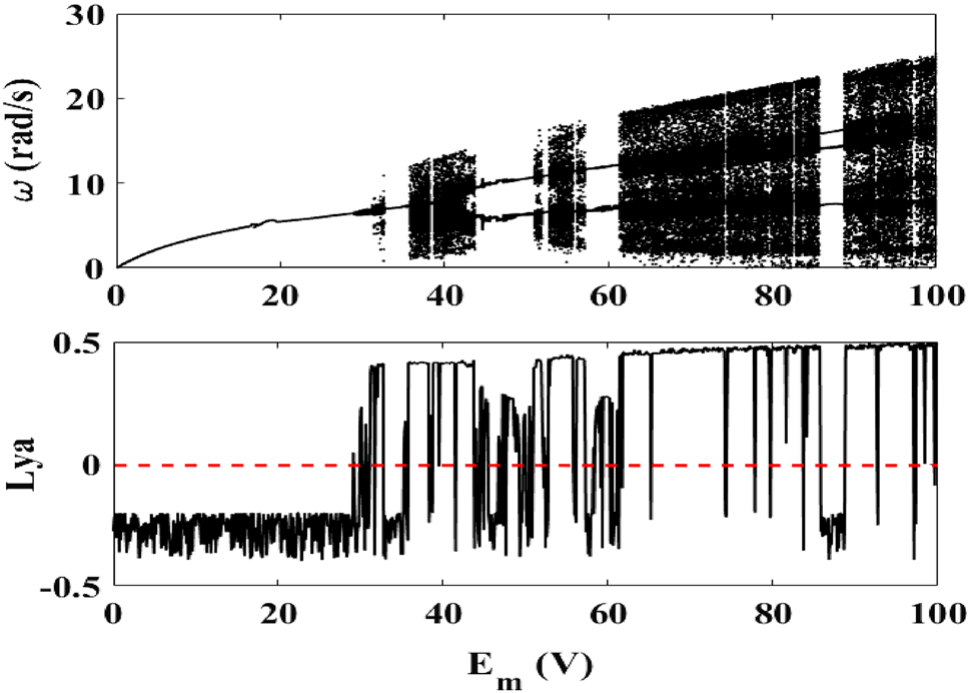
Bifurcation diagram of the motor speed and its Lyapunov exponent for $f = 50\;{\text{Hz}}$, $0\;{\text{V}} \le E_{m} \le 100\;{\text{V}}$, $R_{0} = 5 \, \Omega$ (λ = 11.81 × 10^−2^) and $f_{1} = 2*10^{ - 4} \;{\text{N/m}}^{2}$ (δ_1_ = 1.925).

The frequency of the external source is kept constant at $f$ = 50 Hz with $R_{0} = 5 \, \Omega$. The bifurcation diagram of the motor speed (mechanical part) through the device and the corresponding Lyapunov exponent as function of the generator $E_{m}$ are plotted (see Fig. 4). The bifurcation diagram reveals periodic behavior for small values of $E_{m}$ while chaotic behaviors dominate for large values, which is confirmed by the maximum Lyapunov exponent. Here also, the transitions from periodic behaviors to chaos are abrupt.

### Effect of the frequency $f$

**Figure 5:**
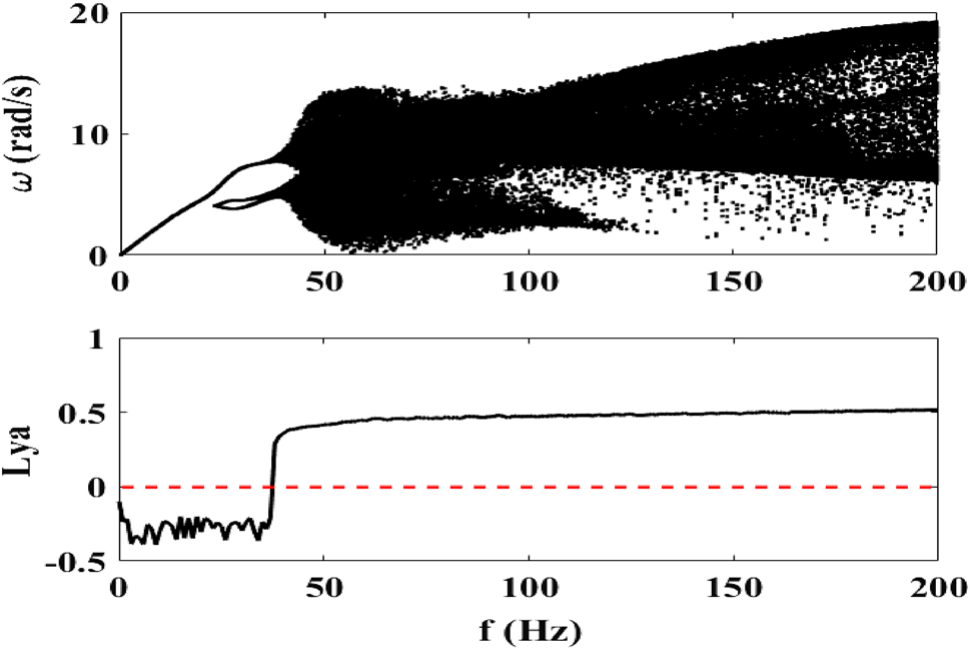
Bifurcation diagram of the motor speed for $E_{m} = 40\;{\text{V}}$ and $0\;{\text{Hz}} \le f \le 200\;{\text{Hz}}$. $R_{0} = 5 \, \Omega$ (λ = 11.81 × 10^−2^) and $f_{1} = 2*10^{ - 4} \;{\text{N/m}}^{2}$ (δ_1_ = 1.925).

Figure 5 shows the bifurcation diagram of the motor speed and its Lyapunov exponent when the frequency $f$ of the excitation external voltage varies. We take $R_{0} = 5 \, \Omega$ and $E_{m} = 40\;{\text{V}}$ in accordance with the preceding bifurcation diagrams and corresponding to the case where the mechanical part exhibits chaotic behavior. It is observed that chaos appears when the frequency is greater or equal than 38 Hz.

### Frequency response curves

**Figure 6:**
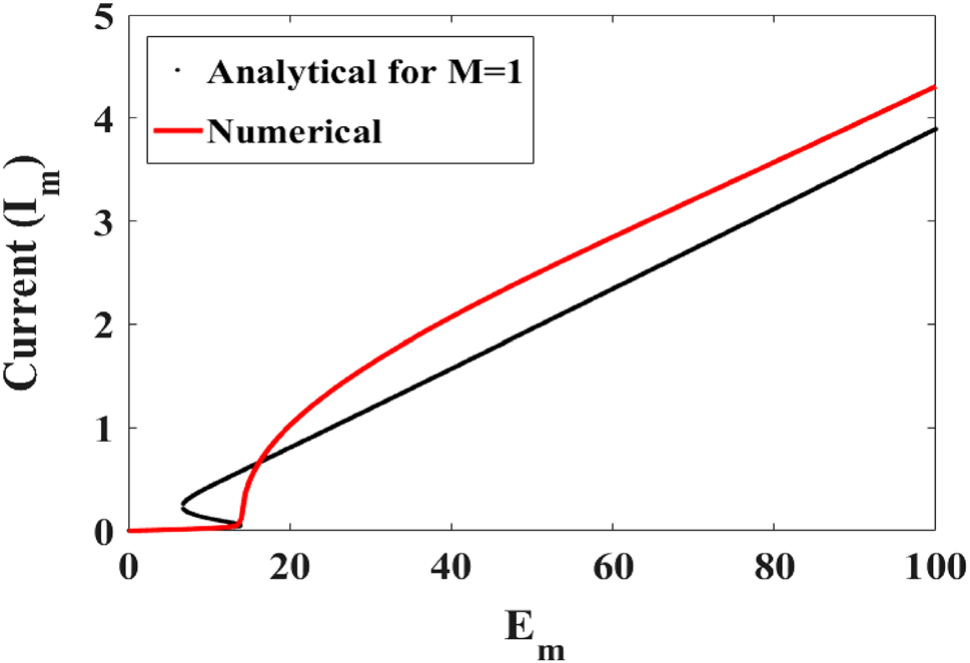
Amplitude of the current versus the magnitude of the external voltage: Analytical at second order (black) and numerical result (red) with $R_{0} = 5 \, \Omega$, $C = 470.32\;{\upmu}$F, $f = 50\;{\text{Hz}}$ and δ_1_ = 1.925.

As it appears in the different bifurcation diagrams, there are domains where the system presents period-1T oscillations. The mathematical expressions of the approximate amplitudes of the period-1T oscillations have been derived in Eq. (13). In this subsection, we compare the amplitude response curves from Eq. (13) to that obtained from the direct numerical simulation of the differential Eqs. (11). For this aim, the capacitance is fixed at $C = 470.32\;{\mu F}$ and the resistance $R_{0} = 5 \, \Omega$. The excitation frequency is fixed at 50 Hz. Figure 6 shows the amplitude of the current versus the magnitude of the external voltage.

One finds that the behavior of the current is nonlinear. For a voltage value between 0 and 6.8 V, the current increases. At 6.8 V, a jump is observed. when the supply voltage is greater than 6.8 V, an increase in the amplitude of the current is observed up to a maximum value of 3.89 A. The phenomenon observed from the curve obtained mathematically is also observed in the results obtained from the numerical simulation (but for a current maximum value reached equal to 4.3 A). There is an agreement between the analytical and numerical results for small values of $E_{m}$. But for large values of $E_{m}$, there is a quantitative difference, although qualitatively the variations of the amplitude as function of $E_{m}$ are quite close.

**Figure 7:**
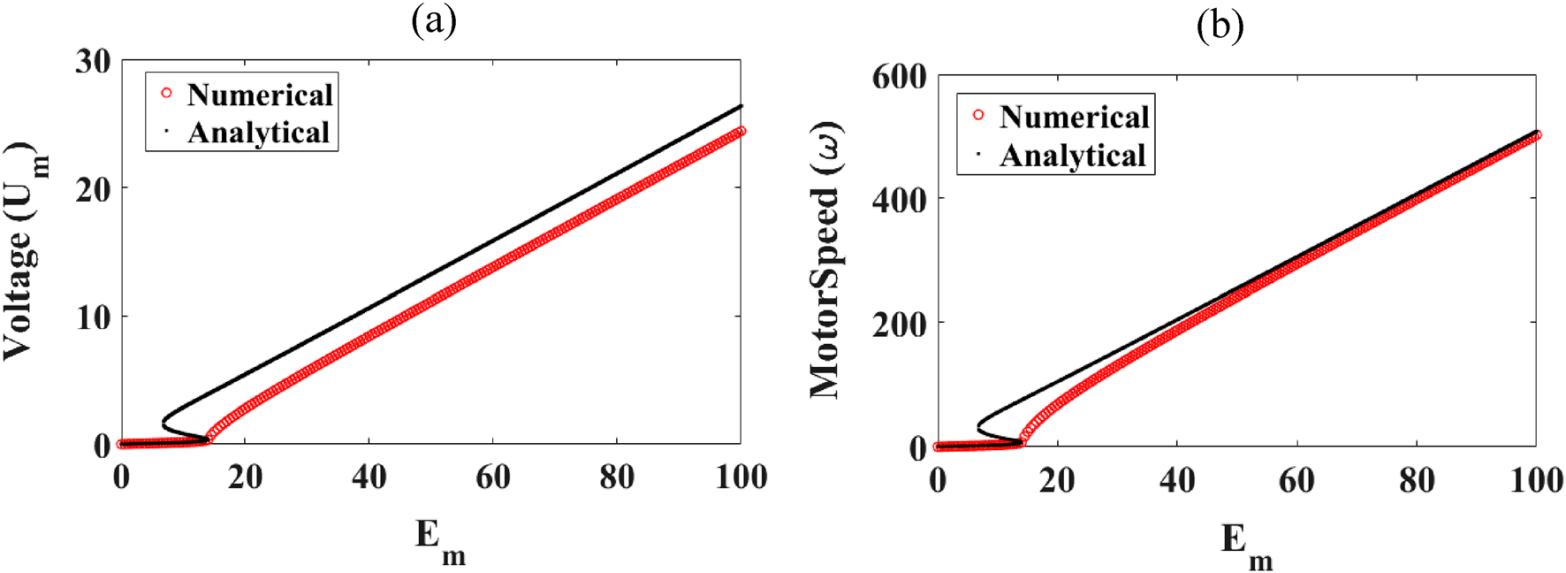
Different amplitudes of the device versus the magnitude of the external voltage: (**a**) the voltage across the capacitor and (**b**) that of the motor speed, with $R_{0} = 5 \, \Omega$, $C = 470.32\;{\mu F}$, $f = 50\;{\text{Hz}}$ and δ_1_ = 1.925.

As for the case of the current, we have plotted the variation of the amplitude of the voltage across the capacitor and that the motor speed versus the amplitude of the excitation voltage. This appears in Fig. 7. We note here that the analytical results are in agreement with the numerical ones for a motor speed ranging between 0 and 13.8 rad/s. For $E_{m}$ greater than 34 V, a good agreement from a qualitative and quantitative point of view is obtained between the numerical results and those obtained from the mathematical derivation.

## Mixer model powered by a square signal

The aim of this section is to study how the mixer behaves when a square voltage is used to power the system. In that case, the equations of motion are given in (15).

15 $$  \left\{ \begin{aligned}    & \left[ {(1 + \eta ) + (1 - \eta )\cosh \left( {x - \sigma } \right)} \right]\dot{x} = \left[ { - \lambda x + \varepsilon y - \gamma z + Esign\left( {\sin (\Omega \tau )} \right)} \right]\left[ {1 + \cosh \left( {x - \sigma } \right)} \right] \\     & \dot{y} =  - qx \\     & \dot{z} =  - \delta _{1} z + \delta _{3} x \\  \end{aligned}  \right.  $$

Here, the frequency and the magnitude of external voltage are used as control parameters. The additional resistance is now set at $R_{0} = 1 \, \Omega$ and the damping coefficient is fixed at δ_1_ = 1.925.

### Effect of the amplitude $E_{m}$

**Figure 8:**
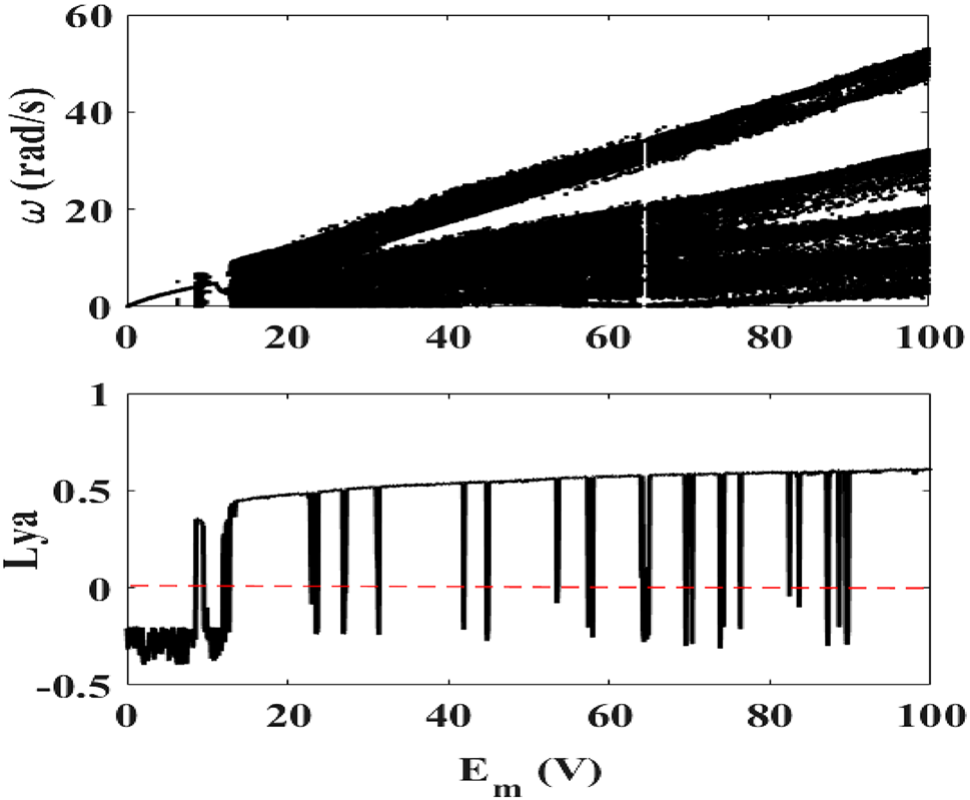
Bifurcation diagram of the motor speed and its maximum Lyapunov exponent for $f$ = 50 Hz and $0\;{\text{V}} \le E_{m} \le 100\;{\text{V}}$.

The frequency of the external voltage source is kept constant at $f$ = 50 Hz. The bifurcation diagram of the mechanical part and its corresponding Lyapunov exponent as a function of the magnitude of the external voltage are shown in Fig. 8. One also finds the domination of chaotic behavior in a large range of the excitation amplitude.

### Effect of the frequency $f$

**Figure 9:**
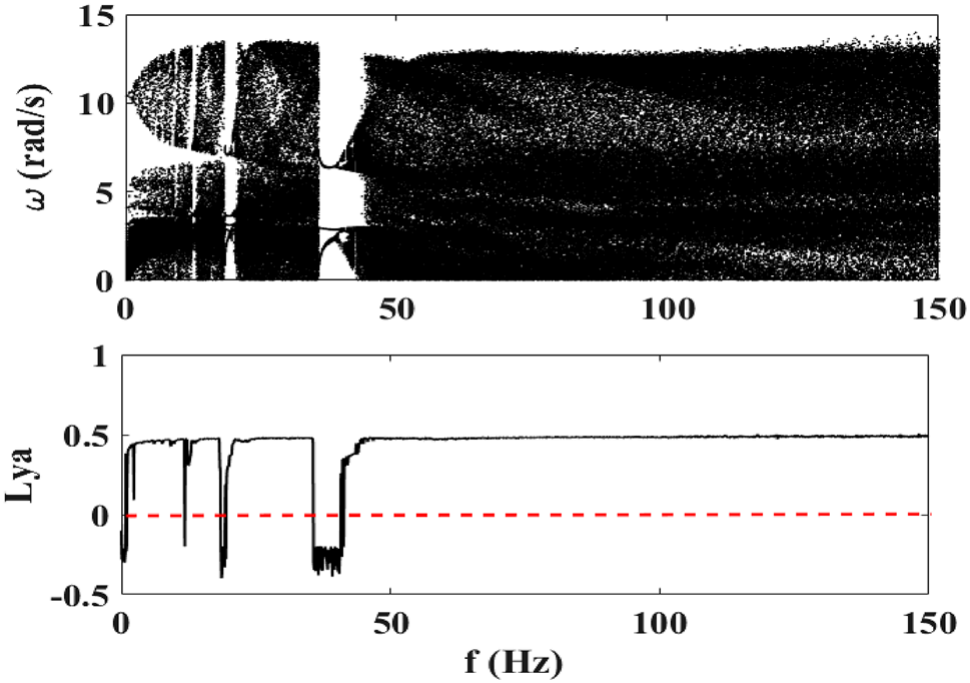
Bifurcation diagram of the motor speed and its maximum Lyapunov exponent for $E_{m} = 20\;{\text{V}}$ and $0\;{\text{Hz}} \le f \le 150\;{\text{Hz}}$.

Considering now the variation of the frequency, Fig. 9 also shows the abundance of chaos in large ranges of the frequency. The frequency varies between 0 and 150 Hz and the amplitude of the voltage source is equal to $E_{m} = 20\;{\text{V}}$. The frequency intervals where chaos is present are as follows: $1.8\;{\text{Hz}} \le f \le 11.6\;{\text{Hz}}$, $13.1\;{\text{Hz}} \le f \le 18.2\;{\text{Hz}}$, $21\;{\text{Hz}} \le f \le 35.5\;{\text{Hz}}$ and $f \ge 44.7\;{\text{Hz}}$.

### Some phase portraits showing chaotic dynamics

In this section, we present the phase portraits showing the voltage across the capacitor and the motor speed respectively as a function of the current flowing through the device. First, we fix the frequency $f$ at 50 Hz and take a value for the amplitude of external voltage source according to the bifurcation diagram of Fig. 8.

**Figure 10:**
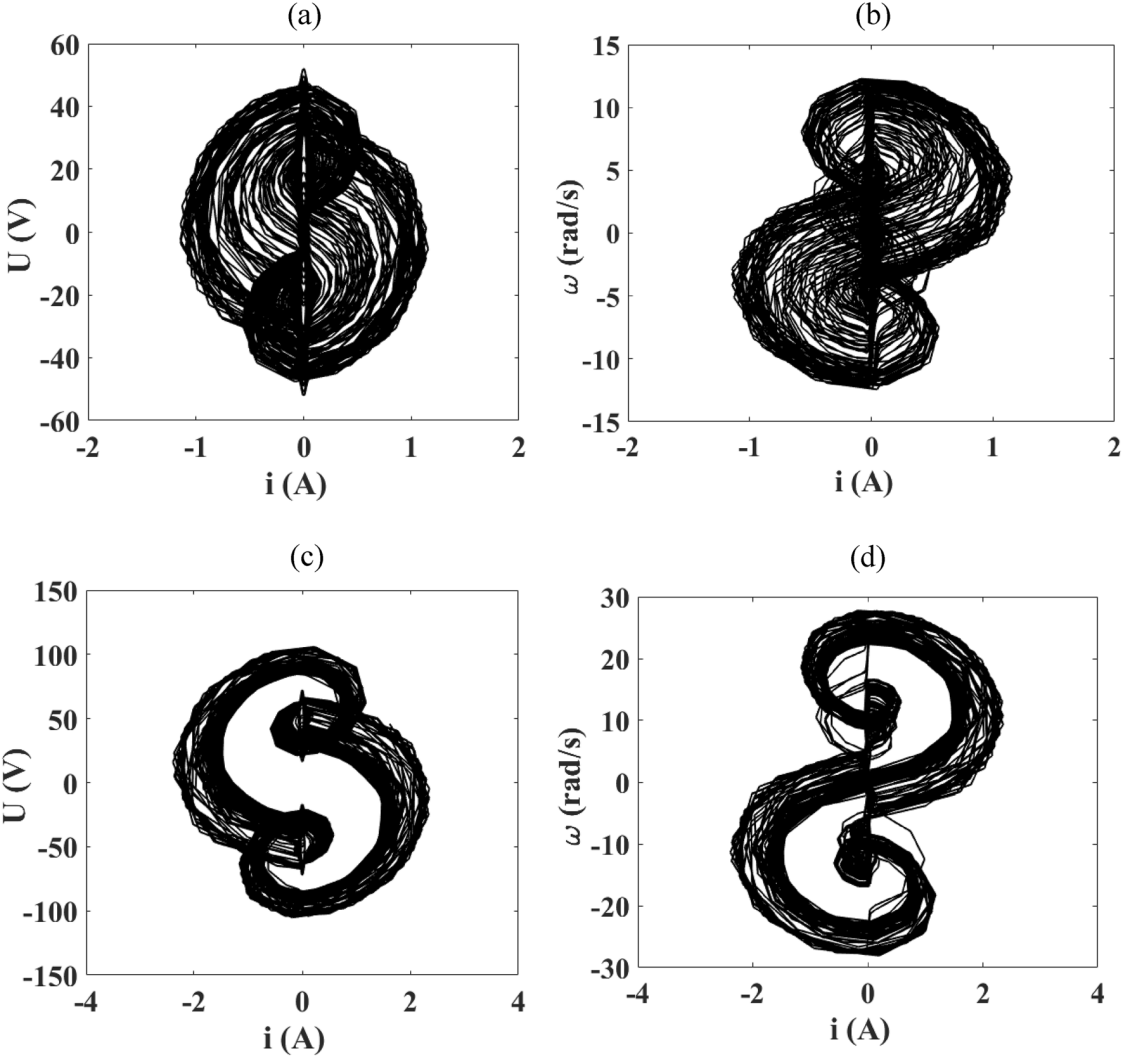
Phase portraits of the device obtained for $f$ = 50 Hz and when the magnitude of the external signal varies: (**a**) and (**b**) for $E_{m} = 20\;{\text{V}}$; (**c**) and (**d**) for $E_{m} = 50\;{\text{V}}$ with $R_{0} = 1 \, \Omega$ and the damping coefficient is fixed at δ_1_ = 1.925.

According to the bifurcation diagram of Fig. 8, we note several chaotic domains when the magnitude of the external force is greater than 12.1 V, which is confirmed by the maximum Lyapunov exponent. We take two values of the magnitude in this range and Fig. 10 presents a chaotic behavior, thus confirming the bifurcation diagram of Fig. 8. We can also note that the intensity of the current flowing through the device increases with the increase of the amplitude of the external voltage source (1.5 A to 3 A). There is also an increase of the voltage across the capacitor and that of the motor speed respectively.

Secondly, we fix the amplitude of the voltage source at $E_{m} = 20\;{\text{V}}$ and take some values of the frequency according to the bifurcation diagram of Fig. 9.

**Figure 11:**
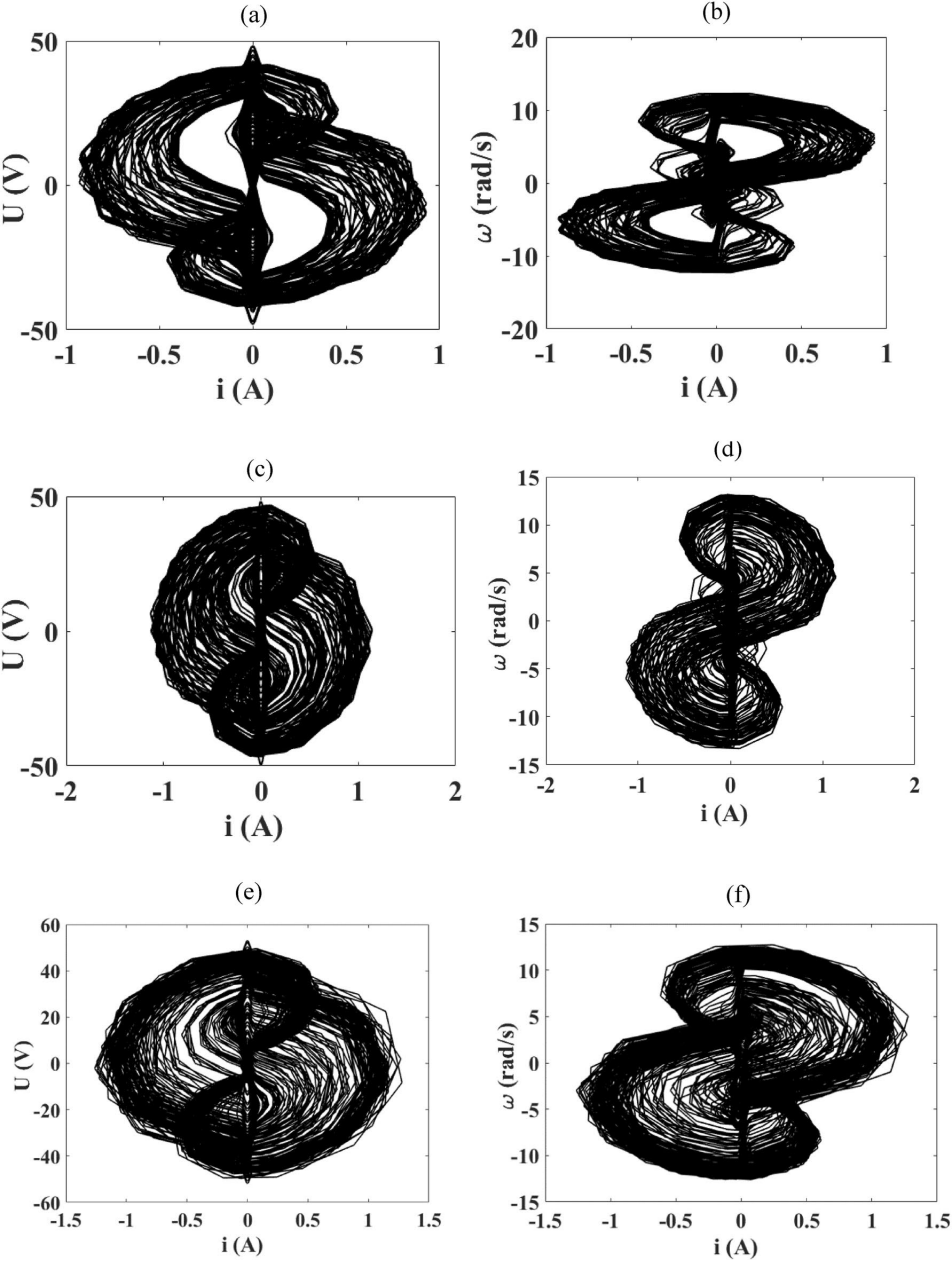
Phase portraits of the device obtained for $E_{m} = 20\;{\text{V}}$ and when the frequency varies: (**a**) and (**b**) for $f$ = 5 Hz; (**c**) and (**d**) for *f* = 35 Hz; (**e**) and (**f**) for *f* = 100 Hz with $R_{0} = 1 \, \Omega$ and the damping coefficient is fixed at δ_1_ = 1.925.

The same observations are also made here as it was the case in Fig. 10. With regards to Fig. 9, we note abundant chaos over almost all frequency ranges. We actually take three frequency values to illustrate this, and Fig. 11 presents the chaotic phase portraits of the device with frequencies taken in intervals presented in the bifurcation diagram of Fig. 9. A maximum current of 1.5 A is obtained under variation of these frequencies.

The increase in current and motor speed when the magnitude of the external signal varies is very significant, especially when homogeneous mixing is required to be carried out with good precision.

### Some times histories showing periodic, chaotic and bursting oscillations

This part first of all discusses the temporal traces of the device when the amplitude of the excitation voltage varies and the frequency is kept at 50 Hz; and secondly when the frequency of the excitation voltage varies and its amplitude is set at 20 V.

**Figure 12:**
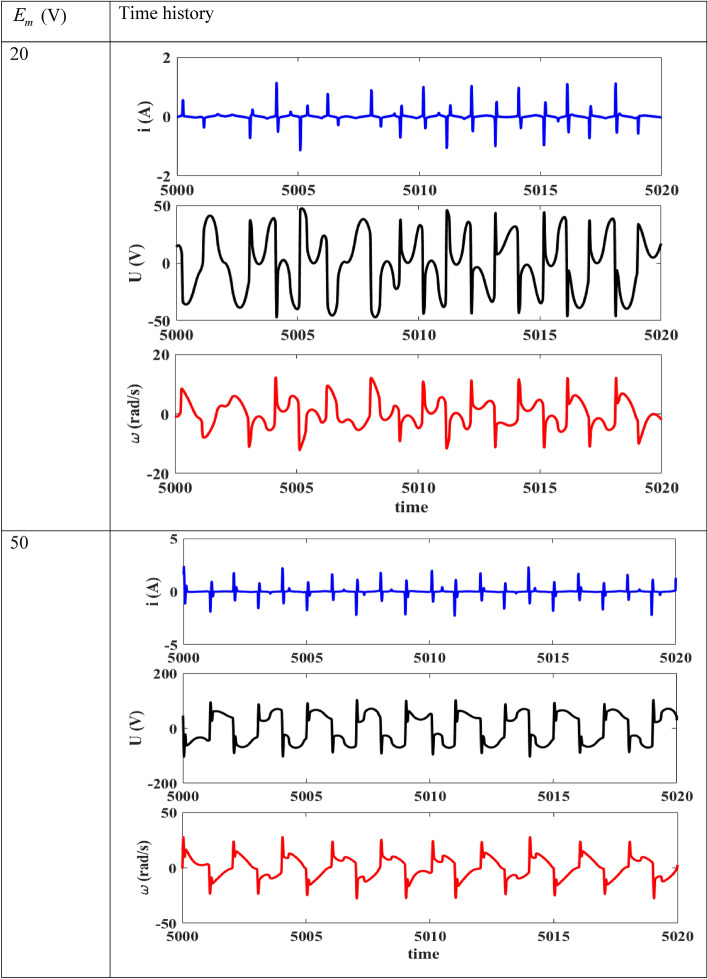
Time histories of the device for $f$ = 50 Hz, $R_{0} = 1 \, \Omega$, δ_1_ = 1.925.

Regarding Fig. 12, we see that for $E_{m} = 20\;{\text{V}}$, the current presents chaotic pulse oscillations. These chaotic behaviors are reproduced respectively at the level of the voltage across the capacitor and of the motor speed. When the amplitude of external voltage source increases, we see that the shape of the oscillations across the capacitor voltage and that of the motor speed changes. The latter exhibits chaotic relaxations.

**Figure 13:**
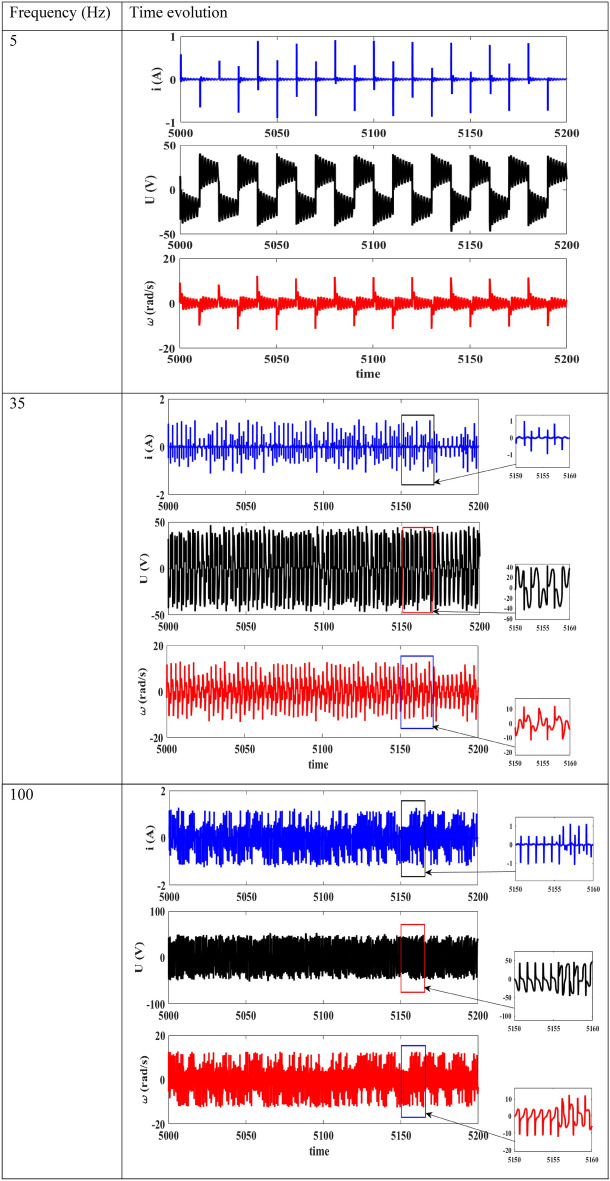
Time histories of the device for $E_{m} = 20\;{\text{V}}$, $R_{0} = 1 \, \Omega$, δ_1_ = 1.925.

With regard to Fig. 13, we see that for $f$ = 5 Hz, the current presents pulse oscillations while the voltage across the capacitor presents bursting oscillations, and the motor speed also presents bursting oscillations. When the frequency increases, there is a decrease of the period of the oscillations and a modification of the shape of the oscillations across the capacitor voltage and that of the motor rotation speed.

Observing figures presented in “Bifurcation diagrams and frequency response curves” and “Mixer model powered by a square signal” section, it can be stated that chaotic behaviors of the RLC circuit are well transferred to the motor at frequency equal to 50 Hz. Such a mixer proposed in this work therefore comes here to improve the domestic mixers that we use at home where we need that the food to be cut be made with a good precision.

## Conclusion

A mixer model with complex rotational movement has been considered in this paper. The device is made up of a nonlinear RLC series circuit with hysteretic iron-core inductor driving a motor. Analytical treatment has been conducted and a good qualitative and quantitative agreement has been found between numerical and analytical results for small values of $E_{m}$. A quantitative difference is observed for high values of $E_{m}$. The numerical simulation of the differential equations of the model has led to the conclusion that chaos is very abundant in the model when one varies the control parameters such as a resistor in the circuit, the damping coefficient due to the resistance created by the type of product to be mixed, the amplitude and frequency of the voltage source. This abundance of chaos is interesting for the envisaged application which is chaotic mixing. The experimental investigation will surely confirm the theoretical results obtained here. Moreover, there is a need to conduct tests for the mixing process and compare the performances of this mixer analyzed here when it runs in chaotic mode and it constant speed mode. Another interesting idea is to create more chaos by using complex mechanical arms can generate turbulent flow during the mixing process. At last, a good point is to understand the impact of chaos in the life time of the motor.

## Data Availability

The datasets used and/or analysed during the current study available from the corresponding author on reasonable request.
